# First clinical expression of equine insect bite hypersensitivity is associated with co-sensitization to multiple *Culicoides* allergens

**DOI:** 10.1371/journal.pone.0257819

**Published:** 2021-11-15

**Authors:** Jasmin Birras, Samuel J. White, Sigridur Jonsdottir, Ella N. Novotny, Anja Ziegler, A. Douglas Wilson, Rebecka Frey, Sigurbjörg Torsteinsdottir, Marcos Alcocer, Eliane Marti

**Affiliations:** 1 Department of Clinical Research and Veterinary Public Health, Vetsuisse Faculty, University of Bern, Bern, Switzerland; 2 School of Animal, Rural and Environmental Sciences, Nottingham Trent University, Brackenhurst Campus, Southwell, United Kingdom; 3 School of Biosciences, University of Nottingham, Loughborough, United Kingdom; 4 Institute for Experimental Pathology, Biomedical Center, University of Iceland, Keldur, Reykjavik, Iceland; 5 Division of Veterinary Pathology, Infection and Immunity, University of Bristol, Langford, United Kingdom; 6 AniCura Norsholms Djursjukhus, Norsholm, Sweden; University of Minnesota, UNITED STATES

## Abstract

**Background:**

Insect bite hypersensitivity (IBH) is an IgE-mediated allergic dermatitis in horses incited by salivary allergens from *Culicoides spp*. IBH does not occur in Iceland, as the causative agents are absent, however a high prevalence is seen in horses exported to *Culicoides*-rich environments.

**Aims:**

To study the natural course of sensitization to *Culicoides* allergens and identify the primary sensitizing allergen(s) in horses exported from Iceland utilizing a comprehensive panel of *Culicoides* recombinant (r-) allergens.

**Method:**

IgE microarray profiling to 27 *Culicoides* r-allergens was conducted on 110 serological samples from horses imported to Switzerland from Iceland that subsequently developed IBH or remained healthy. Furthermore, a longitudinal study of 31 IBH horses determined IgE profiles the summer preceding first clinical signs of IBH (T_IBH_-1), the summer of first clinical signs (T_IBH_) and the following summer (T_IBH_+1). In a group of Icelandic horses residing in Sweden, effects of origin (born in Iceland or Sweden) and duration of IBH (<4 years, 4–7 years, >7 years) on *Culicoides*-specific IgE was evaluated. Sero-positivity rates and IgE levels were compared.

**Results:**

At T_IBH_, horses were sensitized to a median of 11 r-allergens (range = 0–21), of which nine were major allergens. This was significantly higher than T_IBH_-1 (3, 0–16), as well as the healthy (1, 0–14) group. There was no significant increase between T_IBH_ and T_IBH_+1(12, 0–23). IBH-affected horses exported from Iceland had a significantly higher degree of sensitization than those born in Europe, while duration of IBH did not significantly affect degree of sensitization.

**Conclusion:**

Significant sensitization is only detected in serum the year of first clinical signs of IBH. Horses become sensitized simultaneously to multiple *Culicoides* r-allergens, indicating that IgE-reactivity is due to co-sensitization rather than cross-reactivity between *Culicoides* allergens. Nine major first sensitizing r-allergens have been identified, which could be used for preventive allergen immunotherapy.

## Introduction

Insect bite hypersensitivity (IBH), also known as *Culicoides* hypersensitivity, is the most common allergic skin disease in horses. IBH is a seasonal allergic dermatitis caused by hypersensitivity reactions to bites of blood feeding insects of the genus *Culicoides*. Clinical signs are mainly seen in the mane and tail area and derive from severe pruritus which leads to hair loss and excoriations and the development of chronic skin lesions, and sometimes to secondary infections [1, 2].

This disease occurs in all breeds, with a prevalence of 3–10% across Europe. Horses living in Iceland do not suffer from IBH as horse-biting *Culicoides* species are absent. However, >50% of Icelandic horses exported to continental Europe as adults develop IBH within the first 2 years post *Culicoides* exposure, while Icelandic horses born in Europe do not have a higher prevalence of IBH than other breeds [2, 3]. Interestingly, horses exported from Iceland and exposed to *Culicoides* before seven months of age have the same low risk of developing IBH as locally bred horses, suggesting that early exposure to *Culicoides* allergens is essential for the development of immune tolerance [1, 4]. Allergen-specific immunotherapy (AIT) is the only causative treatment for type I hypersensitivities, leading to a shift from a Th2 immune response to a regulatory immune response, in which IgG antibodies are produced that block allergen specific IgE antibodies binding to allergens [5]. Currently, the efficacy of AIT treatment of IBH is questionable, as placebo controlled studies could not demonstrate an effect of AIT compared to the placebo group [6, 7]. This lack of efficacy is most likely due to the fact that crude *Culicoides* whole body extracts were used instead of pure allergens [2]. With the aim to improve AIT and diagnostic serology for IBH, molecular approaches have been applied for the identification of *Culicoides* salivary allergens and their production as recombinant (r-) proteins [2]. Within the last decade, 30 *Culicoides* salivary allergens have been produced as recombinant proteins derived from three *Culicoides* species: *C*. *obsoletus* [8–10] *C*. *nubeculosus* [11, 12] and *C*. *sonorensis* [13]. Microarray profiling of horses from various breeds living in central and northern Europe utilising 27 *C*. *nubeculosus* and *C*. *obsoletus* r-allergens identified nine major *Culicoides* r-allergens, seven of which bound IgE in sera of >70% of IBH-affected horses. The combination of these seven allergens could correctly diagnose >90% of IBH-affected horses with a specificity of ≥95% [10]. All nine major allergens were derived from *C*. *obsoletus*, confirming that allergens derived from *Culicoides* species present in the horse’s environment are more immune-reactive than laboratory-bred species [9, 14].

While the use of AIT is well-established for the treatment of human allergies, its use as preventive immunotherapy in high-risk individuals has been proposed prior to sensitization [15–17]. Whether preventive AIT against IBH is feasible in horses remains to be established, and might become an interesting option to decrease the high incidence of IBH in horses exported from Iceland to continental Europe. However, identification of the primary sensitizing allergens is a prerequisite before such experiments can be performed.

Hence, the objectives of this study were to characterize the natural course of sensitization to *Culicoides* allergens, and identify the primary sensitizing *Culicoides* r-allergen(s) for IBH in horses exported from Iceland to Switzerland. Allergen-specific IgE levels to a large panel of *Culicoides* allergens were determined by protein microarray using sera from a longitudinal study. Additionally, effects of the horse’s origin and duration of IBH (years with IBH) on the pattern of sensitization and levels of allergen-specific IgE were investigated.

## Material and methods

### Horses and blood samples

A total of 224 adult horses of the Icelandic breed were included in the study (Table 1). One hundred and ten horses of which had been exported from Iceland, now residing in Switzerland [18]. Fifty-one horses had remained free from IBH (group H), while 59 developed IBH after export (group IBH). This group was monitored over a period of at least three summers, starting at the time of importation into Switzerland [18]. Longitudinal samples were collected from 31 of the 59 horses i.e. serum IgE levels were analyzed one year before first clinical signs of IBH (T_IBH_-1), the year when IBH occurred for the first time (T_IBH_), as well as the following year (T_IBH_+1). The serum samples from the 51 horses of the H end point group were selected to match the years of sampling in the IBH horses, i.e. the serum samples of the H matched the year of sampling of the IBH-horses at T_IBH_. Forty one of these 51 control horses had been imported the same year as the IBH horses. No longitudinal study was performed on the H end-point group, as a previous study demonstrated there is no increase in allergen-specific IgE in sera from horses with a healthy end-point [19]. Additionally, sera from 22 horses living in Iceland were included in the study. These horses had been used in a previous study [20].

**Table 1:** Horses included in the study.

Living in	Born in Iceland (IS)			Born in continental Europe (N-IS)		Study
	unexposed	IBH	H	IBH	H	
Switzerland (N = 110)	-	59	51	-	-	Torsteinsdottir et al. 2018 [18]
Sweden (N = 92)	-	44	13	11	24	Frey et al. 2008 [20]; Heimann et al. 2011 [21]
Iceland (N = 22)	22	-	-	-	-	Frey et al. 2008 [20]
Total (N = 224)	22	103	64	11	24	

Data and sera from 92 horses living in Sweden (55 with IBH and 37 healthy controls) was also utilized [20, 21]. In 27 of the 55 Swedish IBH horses the duration of disease at time of blood sampling was known, and as such were grouped according to disease duration. Group 1 had clinical signs of IBH for < 4 years, group 2 for 4–7 year, and group 3 for > 7 years.

Horses defined as IBH affected showed the typical recurrent seasonal signs [1], while horses defined as healthy did not show clinical signs of IBH or other skin diseases.

Sera from horses living in continental Europe (exposed to *Culicoides*), affected with insect bite hypersensitivity (IBH) or healthy (H), as well as from healthy horses living in Iceland, thus not exposed to *Culicoides* bites (unexposed). All horses belong to the Icelandic breed. For 31 IBH horses living in Switzerland, data from 3 consecutive time points was available: the year of first clinical signs of IBH (T_IBH_), the preceding (T_IBH_-1) and the following year (T_IBH_+1).

The sera used in the study had been collected between May and November, i.e. during the IBH season. Blood was collected from the jugular vein using Serum Clot Activator-containing vacutainers (Vacuette®; Greiner, St.Gallen, Switzerland). Serum was separated and stored at -80°C until analysis. The study was approved by the Animal Experimental Committee of the Canton of Berne, Switzerland (No. BE 121/05 and BE 2/17). Verbal owner consent was obtained for all horses included in the study.

### Serum IgE profiling by protein microarray

Determination of IgE in serum was performed by protein microarray, as previously described [10, 22, 23], using the same protein microarray as in Novotny et al. [10] which included a total of 27 *Culicoides* r-allergen, *Culicoides* and *Simulium vittatum* extracts, as well as proteins irrelevant for IBH as controls (Table 2).

**Table 2:** Allergens and cut-off values used in the study.

Name	Expression System	Protein family	GenBank	Cut-off used (FAU)
Cul o 1P	Coli	Kunitz Protease Inhibitor	JX512273	7300
Cul o 2	Baculo	Hyaluronidase	KC339672	150
Cul o 2P	Coli	D7-related/OBP	JX512274	300
Cul o 3	Coli	PR1 like (antigen-5 like)	KC339673	1070
Cul o 3P	Coli	D7-related/OBP	JX512275	870
Cul o 5	Coli	Unknown	KC339675	6000
Cul o 6	Pichia	D7-related / OBP	KC339676	238
Cul o 7	Baculo	Unknown	KC339677	900
Cul o 8	Coli	Kunitz protease inhibitor	MN123710	1650
Cul o 9	Coli	WSC superfamily, carbohydrate binding domain	MN123712	1800
Cul o 10	Coli	DUF4803 superfamily	MN123711	700
Cul o 11	Coli	Apolipophorin III like	MN123713	11000
Cul o 12	Coli	Leucin rich repeat	MN123714	488
Cul o 13	Coli	D7-related/OBP	MN123715	5600
Cul o 14	Coli	Serine protease/Trypsin	MN123716	160
Cul o 15	Coli	Apyrase	MN123717	1100
Cul n 1	Baculo	PR1 like (antigen-5 like)	EU978899	2500
Cul n 2	Baculo	Hyaluronidase	HM145950	230
Cul n 3	Baculo	†DUF4803 superfamily	HM145951.1	7500
Cul n 4	Barley	Unknown	HM145952	820
Cul n 5	Coli	DUF4803 superfamily	HM145953	108
Cul n 6	Coli	unknown	HM145954	800
Cul n 7	Coli	Unknown	HM145955	2200
Cul n 8	Baculo	Maltase (alpha amylase)	HM145956	3950
Cul n 9	Coli	D7-related/OBP	HM145957	2400
Cul n 10	Coli	DUF4803 superfamily	HM145958	3500
Cul n 11	Coli	Serine Protease/Trypsin	HM145959	1500
CN-TE	extract		[19]	1150
SV-WBE	extract		[18]	1800
CO-WBE	extract			1550
Alt a 1	Coli		Biomay	300
Der f	extract		StallergeneGreer	3700

These proteins had been normalized to 0.5 mg/ml protein and printed onto Grace Bio-Labs Oncyte® Nova™ nitrocellulose film slides using a Marathon microarrayer (Arrayjet, Roslin, Scotland) [22]. Slides were first blocked with 3% BSA in PBS. Sera, diluted 1:2, were applied and the slides hybridized O/N at 4°C. After washing, anti-horse IgE mAb 3H10 [24] was added and incubated at 37°C for 2h, followed by an incubation with DyLight 649 conjugated anti-mouse IgG1 for 1h (Rockland, #610-443-040). The slides were then dried via centrifugation and scanned using a GenePix4000B (Molecular Devices, Inc., Sunnyvale, CA, USA). For each protein blank values (obtained by adding all reagents except serum) and background fluorescence were subtracted from the values obtained with the sera before further analyses of the data. Data were presented as fluorescence arbitrary unit (FAU).

*Culicoides nubeculosus* (Cul n) and *Culicoides obsoletus* (Cul o) recombinant allergens and Cul n thorax extract (CN-TE), Cul o group whole body extract (CO-WBE) and *Simulium vittatum* whole body extract (SV-WBE) used in the study. Cut-off values used (in fluorescence arbitrary units, FAU) are those defined previously [10]. *Alternaria alternate* 1 (Alt a 1) and *Dermatophagoides farinae* (Der f) were used as control proteins not relevant for IBH.

### Statistical analyses

For statistical analyses, NCSS software (NCSS 12 Statistical Software (2018) NCSS, LLC. Kaysville, Utah, USA, ncss.com/software/ncss) was used. Since the data was not normally distributed, descriptive statistics using median and ranges were used. The non-parametric Kruskal-Wallis Multiple-Comparison Z-Value test (Dunn’s test) with Bonferroni correction for multiple comparisons was used to analyze differences in allergen-specific IgE concentrations or numbers of positive IgE values between IBH-affected, healthy control and unexposed horse groups as well as between horses born in Iceland (IS) and born in continental Europe (N-IS), and horses grouped according to the duration of IBH.

For each allergen, specific IgE values were transformed to positive and negative (above and below cut-off level) results. As IgE measurement of these samples was carried out within few weeks of those from Novotny et al. [10], the same cut-off values were used (Table 2). Values giving at least a specificity of 94% at the highest accuracy possible had been selected as cut-offs [10].

Median values between time points in the longitudinal study were compared using the non-parametric paired Wilcoxon Signed-Rank Test. Bonferroni correction for multiple comparisons (cP) was performed manually (P-value x number of comparisons = cP).

The 2-sided Fisher’s exact test was used to compare the proportion of IBH-affected, H horses and unexposed horses with positive allergen-specific IgE results, or to compare the proportion of positive results in horses born in Iceland and exported to Sweden or born and living in Sweden. When multiple comparisons were performed, cP was used as mentioned above. P ≤ 0.05 was regarded as significant throughout the paper.

## Results

### Allergen-specific IgE in sera from horses born in Iceland and imported to Switzerland

First, IgE levels to *Culicoides* allergens in sera collected the first summer of clinical onset of IBH were compared between the IBH (n = 59) and the H group (n = 51), as well as unexposed horses that were living in Iceland (n = 22).

**Fig 1:**
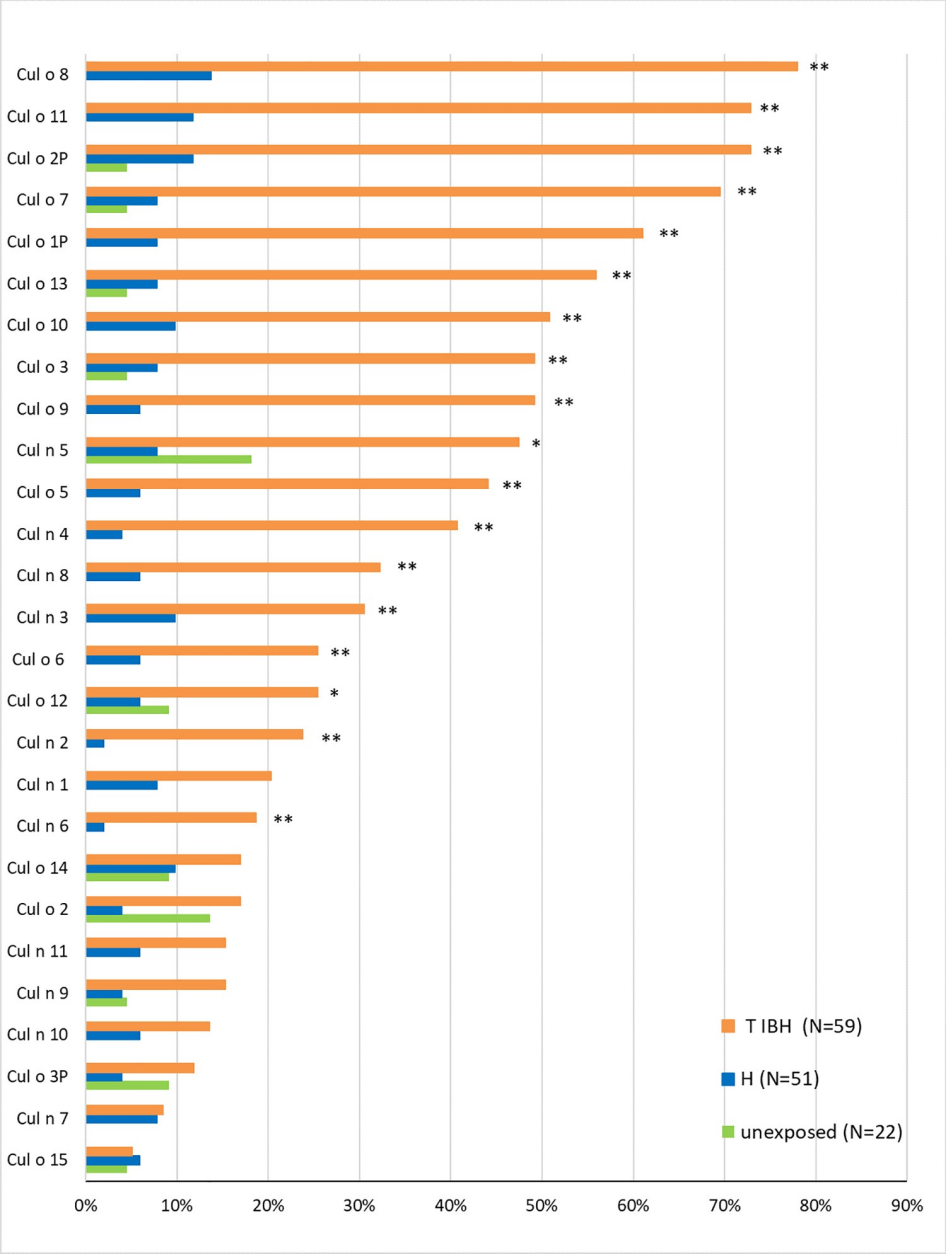
IgE sero-positivity to 27 *Culicoides* recombinant (r-) allergens. Percentage of horses with IgE levels above the cut-off values (as indicated in Table 2) in horses exported from Iceland to Switzerland that developed insect bite hypersensitivity (IBH; n = 59) or remained healthy (H; n = 51), and in horses living in Iceland (unexposed; n = 22). Serum samples were taken the summer of clinical onset of IBH (T_IBH_) and at the corresponding time in the H group. The allergens are listed in decreasing order from those binding serum IgE in the highest number of horses at time of clinical onset of IBH (T_IBH_). P values were calculated with the Fisher’s exact test and Bonferroni correction done for multiple comparisons. ** IBH significantly different from H and unexposed (cP <0.05). * IBH significantly different from H (cP<0.05). No significant differences between H and unexposed for any r-allergen.

Depending on the *Culicoides* r-allergen, the percentage of IBH horses with positive IgE values ranged from 5 to 78%. In the H group, 2 to 14% of the horses had positive IgE values, and in the unexposed horses between 0 to 18% (Fig 1). There were no significant differences between the unexposed and H groups for any of the tested r-allergens. Surprisingly, there were some IgE values above cut off in the unexposed horses, however IgE concentrations were usually low (S1 Table). Eighteen of the 27 tested *Culicoides* r-allergens bound serum IgE in a significantly higher percentage of IBH-affected horses compared to the H horses (Fig 1). Seven of these allergens (Cul o 8, Cul o 11, Cul o 2P, Cul o 7, Cul o 1P, Cul o 13 and Cul o 10) bound serum IgE in >50% of the IBH-affected horses, and two in almost 50% of them (Cul o 3 and Cul o 9, each 49.2%). Fig 1 shows that the allergens Cul o 8, Cul o 11 and Cul o 2P bound serum IgE in > 70% of the IBH affected horses, Cul o 7 and Cul o 1P in > 60% and Cul o 13 and Cul o 10 in >50%.

Median IgE levels to the r-*Culicoides* allergens in the three groups of horses are shown in S1 Table. Interestingly, while there were no significant differences between H and IBH-horses for IgE concentrations specific for the irrelevant proteins (Der f and Alt a 1), the horses living in Iceland (unexposed) had significantly higher IgE to these allergens than those living in Switzerland.

### Longitudinal study of allergen-specific IgE in sera from horses that developed IBH

**Fig 2:**
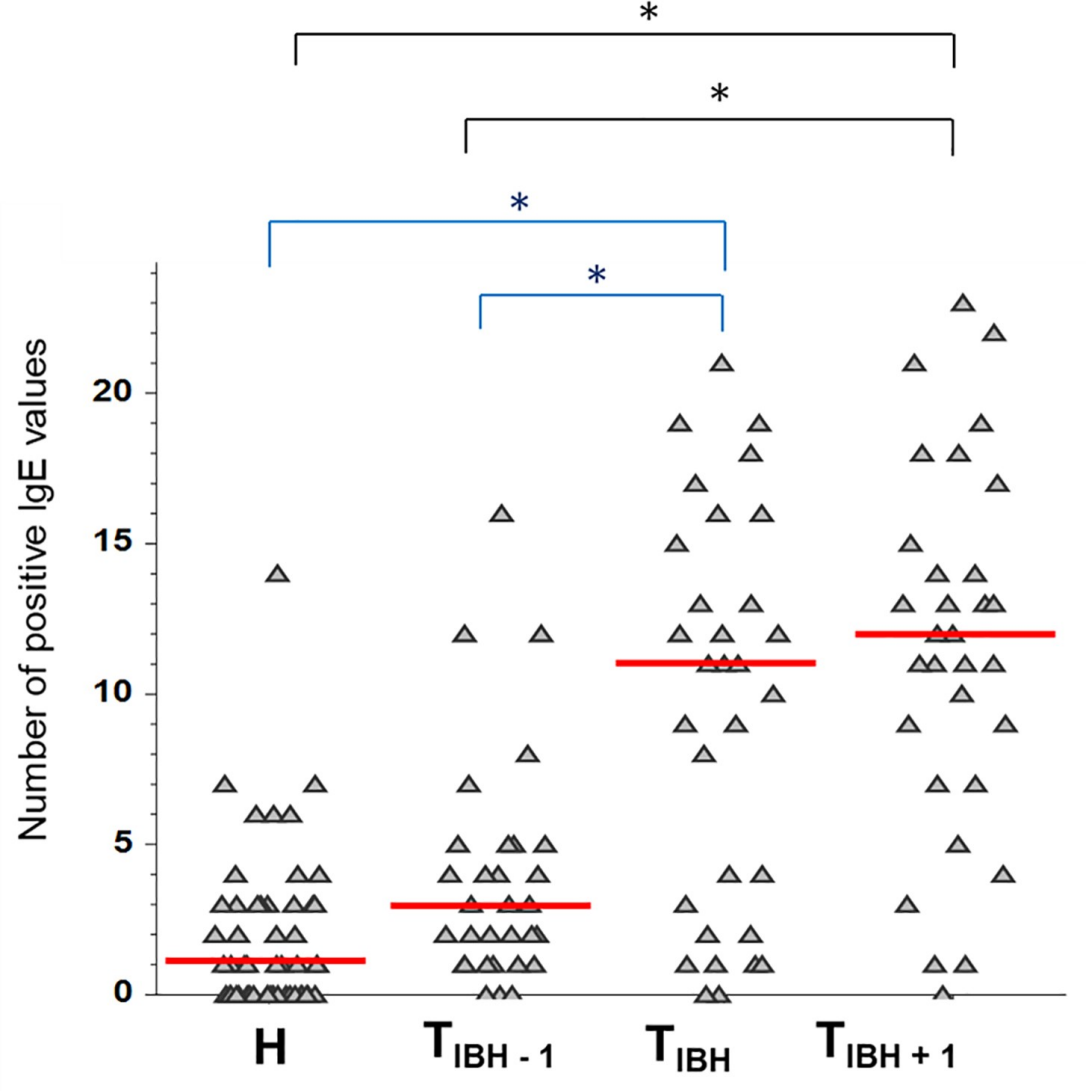
Cumulative number of positive serum IgE values per horse for the 27 r-allergens at time of clinical onset of IBH (T_IBH_), one year before (T_IBH_-1) and one year after (T_IBH_+1) and in healthy control horses (H). Each symbol represents a separate horse and red lines represent the medians. The Kruskal-Wallis Multiple-Comparison Z-Value test (Dunn’s test) with Bonferroni correction for multiple comparisons was used. * indicates significant differences between the time points (P ≤ 0.01).

In a subgroup of 31 horses that developed IBH following import, IgE reactivity patterns to *Culicoides* r-allergens were determined at three different time points: the year preceding first clinical signs of IBH (T_IBH_-1), the year when IBH occurred for the first time (T_IBH_) and the following year (T_IBH_-1). The number of positive IgE reactions to *Culicoides* r-allergens increased significantly between T_IBH_-1 and T_IBH_, from a median number of three (range 0–16) to eleven positive reactions (range 0–21), while there was no significant increase between T_IBH_ and T_IBH_+1. At this last time point, IBH horses had positive IgE values to a median number of 12 (range 0–23) *Culicoides* r-allergens (Fig 2).

**Fig 3:**
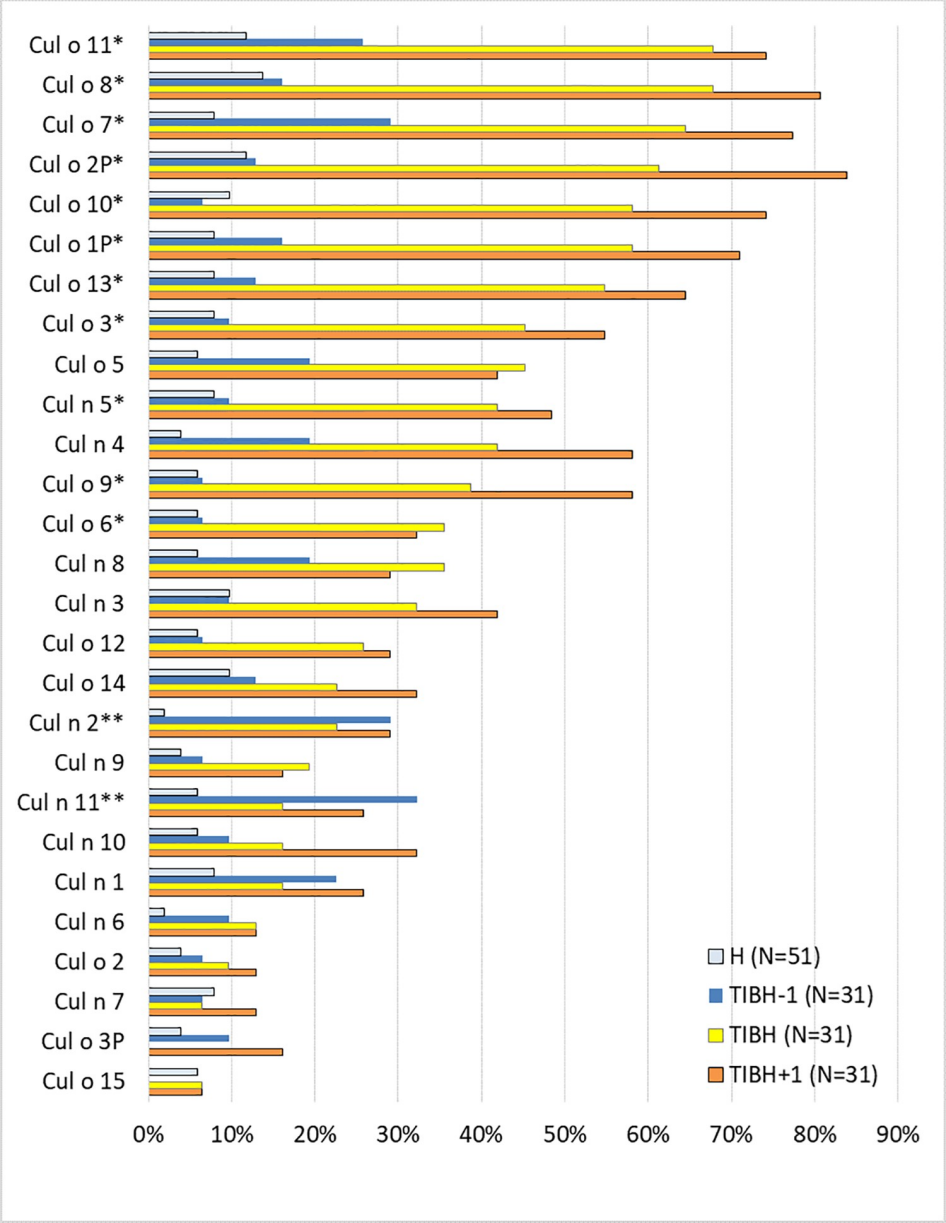
Percentage of horses with IgE levels above the cut-off values following import from Iceland to Switzerland. Horses with IgE levels above the cut-off values (as indicated in Table 2) at time of clinical onset of IBH (T_IBH_), one year before (T_IBH_-1)) and one year after (T_IBH_+1) and in healthy horses for comparison. P values were calculated with the Fisher’s exact test and Bonferroni correction done for multiple comparisons. * significant difference between T_IBH_ and T_IBH-1_ (cP < 0.05). ** significant difference between T_IBH_-1 and H (cP < 0.05).

At T_IBH_-1, horses that developed IBH did not show significantly higher sero-positivity rates to individual r-allergens than the H group, except for Cul n 2 and Cul n 11 (Fig 3). However, for both of these allergens the sero-positivity rate remained low over time (<33%).

Analysis of the reactivity to the individual *Culicoides* r-allergens demonstrates that the number of positive IgE reactions increased significantly between T_IBH_-1 and T_IBH_ for ten Cul o r-allergens, as well as for Cul n 5 (Fig 3). At T_IBH_, the allergens Cul o 11, 8, 7, 2P, 10, 1P and 13 bound IgE in >50% of the IBH sera. Even though comparison of the percentage of IgE positive reaction between T_IBH_ and T_IBH_+1 did not reach statistical significance for any *Culicoides* r-allergen, for many r-allergens a further increase in the number IgE positive horses was observed. At T_IBH_+1 >80% of the horses had positive IgE values with Cul o 8 and Cul 2P, >70% with Cul o 11, Cul o 7, Cul o 10 and Cul o 1P and >60% with Cul o 13.

IgE levels were compared using a paired T-test to test whether the amount of free serum IgE to a given allergen increased in the single horses over time (Table 3). As expected from the previous analysis (Fig 3), for most r-allergens the main increase in IgE levels was observed between T_IBH_-1 and T_IBH_ (Table 3). Between T_IBH_ and T_IBH_+1 a significant increase in IgE levels was observed only for Cul o 1P, Cul o 8, Cul o 9 and Cul n 10.

**Table 3:** Median serum IgE levels to *Culicoides* recombinant (r-) allergens.

Allergen name	T_IBH_-1 (n = 31)		T_IBH_ (n = 31)		T_IBH_+1 (n = 31)	
	median	range	median	range	median	range
Cul o 1P	**1871** ^**a**^	0–50731	**13269** ^**a**^ ^,^ ^**b**^	0–59314	**45226** ^**b**^	0–55123
Cul o 2	600	0–179	944	0–763	1338	0–263
Cul o 2P	**52** ^**a**^	0–3143	**471** ^**a**^	0–23064	1214	0–8783
Cul o 3	**332** ^**a**^	34–5643	**905** ^**a**^	82–18971	1149	210–6384
Cul o 3P	118	0–2031	103	0–1584	189	0–21848
Cul o 5	2655	0–49677	4937	564–55803	5690	419–56749
Cul o 6	**43** ^**a**^	0–1782	**91** ^**a**^	0–3603	134	0–2031
Cul o 7	**353** ^**a**^	64–43021	**2148** ^**a**^	111–50627	4457	77–60346
Cul o 8	**3940** ^**a**^	0–57708	**46484** ^**a**^ ^,^ ^**b**^	0–60605	**48671** ^**b**^	2479–60389
Cul o 9	**59** ^**a**^	0–50264	**485** ^**a**^ ^**,b**^	0–61076	**3988** ^**b**^	0–63029
Cul o 10	**32** ^**a**^	0–3311	**1220** ^**a**^	0–48437	1952	0–49432
Cul o 11	**7267** ^**a**^	1457–47315	**29123** ^**a**^	1035–58052	26223	1534–59117
Cul o 12	**11** ^**a**^	0–528	**76** ^**a**^	0–5598	249	0–2817
Cul o 13	**1825** ^**a**^	150–34346	**6641** ^**a**^	61–48342	8935	318–53397
Cul o 14	0	0–347	29	0–1062	36	0–2493
Cul o 15	**66** ^**a**^	0–863	**135** ^**a**^	0–5573	166	0–1310
Cul n 1	728	0–12989	590	0–40387	1007	0–45461
Cul n 2	108	0–530	68	0–3049	90	0–2536
Cul n 3	**672** ^**a**^	100–29127	**3349** ^**a**^	101–59729	4763	85–57789
Cul n 4	**70** ^**a**^	0–33717	**531** ^**a**^	0–37001	1329	0–31529
Cul n 5	**32** ^**a**^	0–218	**79** ^**a**^	0–4296	97	0–1515
Cul n 6	102	0–3805	45	0–2087	54	0–45799
Cul n 7	478	0–5488	351	0–5495	361	0–7782
Cul n 8	1185	165–9676	1856	108–15927	1595	21–16744
Cul n 9	619	176–18617	749	38–9550	798	0–27699
Cul n 10	944	0–6466	**664** ^**b**^	6–55321	**1403** ^**b**^	0–35385
Cul n 11	498	0–10235	315	0–9431	532	0–17096

Median serum IgE levels (in fluorescence arbitrary units) to *Culicoides* recombinant (r-) allergens in horses imported from Iceland to Switzerland that developed insect bite hypersensitivity (IBH). Median IgE values in sera taken one year before 1^st^ clinical signs of IBH (T_IBH_-1), the year when clinical signs of IBH were first observed (T_IBH_) and the following year (T_IBH_+1) were compared using the non-parametric paired Wilcoxon Signed-Rank Test and Bonferroni corrections for multiple comparisons (cP).

^**a**^ Significant difference between T_IBH_-1 and T_IBH_.

^**b**^ Significant difference between T_IBH_ and T_IBH_+1.

### Comparison of allergen-specific IgE in sera of horses from the Icelandic breed with different origins

The influence of the origin of the horse (i.e. born in Iceland and exported to Sweden or born in Sweden) on *Culicoides* r-allergen specific IgE was evaluated in a group of horses located in Sweden. In horses born in Iceland and exported to Sweden, IBH-affected horses had significantly higher median IgE levels to 20 different *Culicoides* r-allergens compared to H horses. Within horses born in Sweden, IBH-affected horses had significantly higher IgE levels to eight of these *Culicoides* r-allergens (Table 4). Moreover, IgE levels did not differ significantly between H horses born in Sweden or H horses born in Iceland and exported to Sweden. Horses exported from Iceland that later developed IBH had significantly higher median IgE levels to Cul o 6, Cul o13 and Cul n 5 than IBH horses born in Sweden. Particularly Cul o 13 seems to be of high importance in IBH-affected Iceland-born horses, while not in continental-born IBH horses.

**Table 4:** Effect of the origin of the horse on median values of *Culicoides*-specific IgE levels.

Allergen	IS-H (N = 13)		IS-IBH (N = 44)		N-IS-H (N = 24)		N-IS-IBH (N = 11)		
	median	range	median	range	median	range	median		range
Cul o 1P	491	0–14889	47128	169–59261	906	0–7029	42471	4026–52188	
Cul o 2	35	0–234	168	4–6112	35	0–146	36	0–2230	
Cul o 2P	53	0–199	3387	37–53231	20	0–429	561	108–15757	
Cul o 3	195	32–1067	1837	125–24957	184	25–2169	526	172–5540	
Cul o 3P	446	47–3523	913	48–51070	310	0–1537	610	0–2646	
Cul o 5	523	237–22917	16522	258–53700	1048	75–8382	8104	574–47024	
Cul o 6	91	0–417	**313** ^**a**^	0–12676	46	0–376	**66** ^**a**^	0–14442	
Cul o 7	183	18–582	15591	60–57396	358	33–2492	1795	244–15408	
Cul o 8	3625	0–13227	46296	3647–55048	1125	46–10171	42138	1102–50688	
Cul o 9	76	0–1833	40984	38–56877	57	0–978	10795	52–53911	
Cul o 10	116	0–1233	20245	0–55174	204	0–612	9215	107–44976	
Cul o 11	2608	667–34063	41605	2679–54109	1722	380–8559	28932	8193–46491	
Cul o 12	20	0–1017	1317	0–25360	8	0–774	545	0–13677	
Cul o 13	883	74–22480	**9167** ^**a**^	838–49726	700	31–3226	**1121** ^**a**^	123–16088	
Cul o 14	18	0–114	190	0–2837	23	0–354	38	0–2214	
Cul o 15	76	5–513	233	13–13921	68	0–1069	171	0–5898	
Cul n 1	381	1–3269	1329	184–52050	426	65–2790	831	0–48398	
Cul n 2	96	0–430	234	0–4793	50	0–363	67	0–1023	
Cul n 3	1119	211–2192	11763	344–56358	1499	134–24146	5486	862–12438	
Cul n 4	81	0–556	2266	121–54464	152	0–871	468	0–17197	
Cul n 5	54	0–117	**227** ^**a**^	0–4777	9	0–108	**24** ^**a**^	0–179	
Cul n 6	228	0–2142	746	0–38382	180	0–1406	412	0–2303	
Cul n 7	659	0–4642	462	0–23991	292	0–4447	242	0–983	
Cul n 8	967	139–3462	1173	5–19481	1120	0–8281	1788	331–9573	
Cul n 9	660	71–5671	1824	137–51165	457	158–13932	2159	178–21507	
Cul n 10	658	0–11781	1374	0–49574	477	0–5097	334	57–9265	
Cul n 11	174	0–2461	877	0–42727	292	0–1658	210	0–24667	
CO-WBE	549	205–2124	2321	301–36169	482	31–1860	1703	485–30119	
CN-TE	744	192–2550	1553	198–42326	464	55–1981	1498	476–7854	

Effect of horse origin (born in Iceland and exported to Sweden (IS) or born in Sweden and living in Sweden (N-IS)) on median values of *Culicoides*-specific IgE levels (in fluoresence arbitrary unit) in IBH-affected and healthy (H) horses. The Kruskal-Wallis Multiple-Comparison Z-Value test (Dunn’s test) with Bonferroni correction was used to analyze differences in allergen-specific IgE levels.

Pink: Significant difference between healthy and IBH IS horses.

light blue: Significant difference between healthy and IBH N-IS horses.

^**a**^ Significant difference between IS-IBH and N-IS IBH horses.

No significant differences were observed between IS-H and N-IS-H horses.

### Effect of duration of IBH on allergen-specific IgE levels

**Fig 4:**
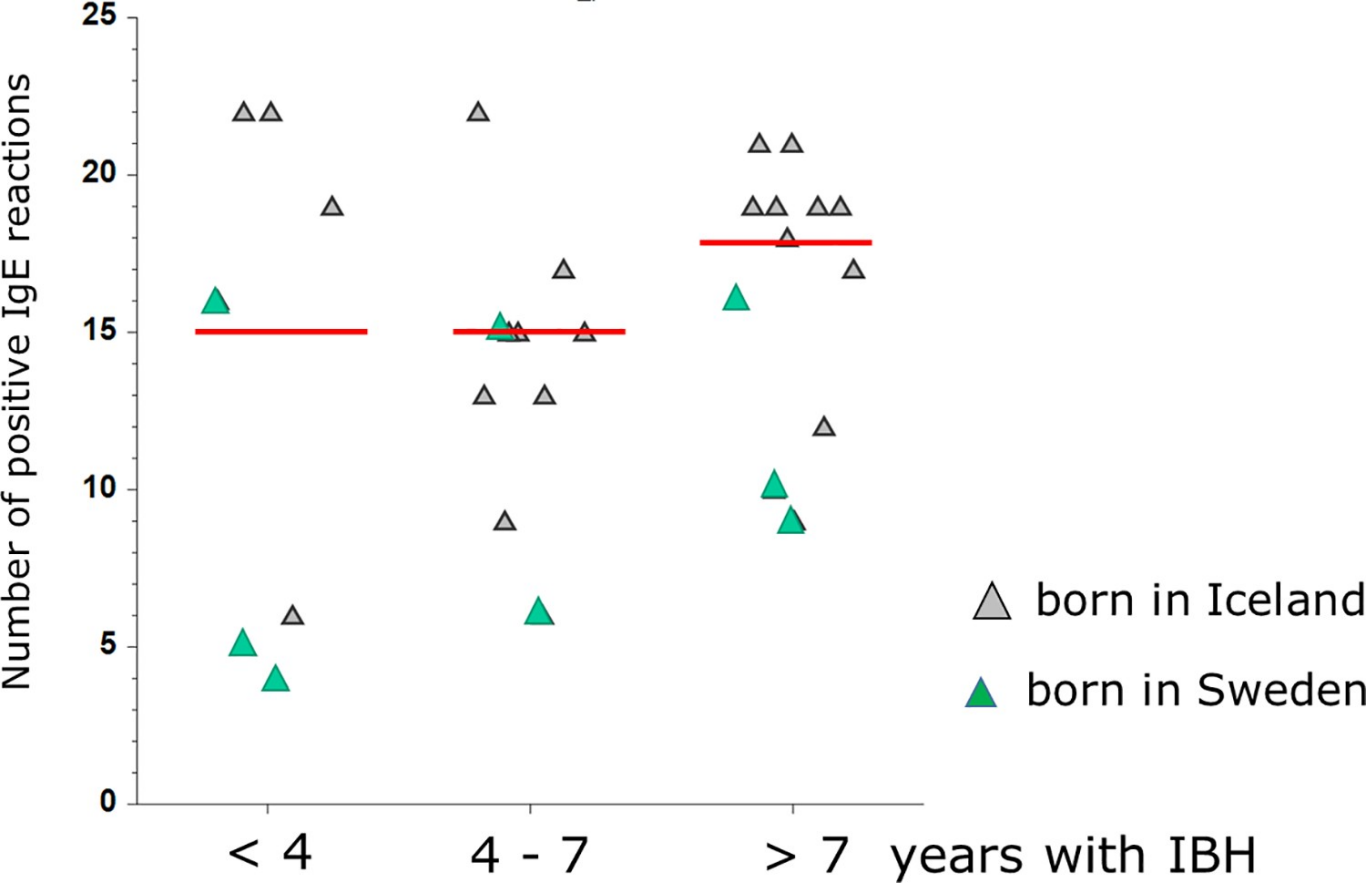
Effect of duration of IBH on cumulative number of positive serum IgE values. Icelandic horses living in Sweden grouped according to how long they had been affected with IBH (< 4 years, 4–7 years or >7 years). No significant differences between the groups in the Kruskal-Wallis Multiple-Comparison Z-Value test. Green triangles identify horses that were born in Sweden and grey triangles those that were born in Iceland and exported to Sweden.

To investigate whether horses affected with IBH for several years have higher IgE levels and react to a higher number of r-allergens, IBH-affected horses living in Sweden were grouped according to disease duration. Horses suffering from IBH for <4 years had IgE positive to a median number of 15 r-allergens (range 4–22). This value remained the same in horses affected with IBH for a duration of 4 to 7 years (median 15, range 6–22) and increased to 18 (range 8–21) r-allergens for horses suffering from IBH for more than 7 years, but this difference was not significant (Fig 4).

Comparison of IgE concentrations between the groups showed that for most r-allergens IgE levels do not differ significantly depending on duration of IBH. Nevertheless, horses with a long duration of IBH (>7 years) have significantly higher IgE levels to Cul o 1P, Cul o 5 and Cul o 10 compared with horses suffering from IBH for <4 years (Table 5).

**Table 5:** Effect of duration of IBH on median IgE levels to *Culicoides* r-allergens.

Allergen	IBH < 4 years (N = 7)		IBH 4–7 years (N = 9)		IBH > 7 years (N = 13)	
	median	range	median	range	median	range
Cul o 1P	**27020** ^**a,b**^	664–51471	**49206** ^**a**^	42471–59261	**48674** ^**b**^	41046–56746
Cul o 2	234	0–6112	87	0–660	270	0–3949
Cul o 2P	387	108–5277	657	70–53231	2364	185–13293
Cul o 3	2092	344–7757	938	172–10148	1732	285–24957
Cul o 3P	968	0–5997	559	0–2521	1096	569–20970
Cul o 5	**5984** ^**b**^	1748–32944	27955	1385–47024	**30804** ^**b**^	1890–49394
Cul o 6	316	4–14442	515	0–3086	310	9–5993
Cul o 7	2289	946–55017	5658	721–41237	26305	244–53549
Cul o 8	42747	3647–47801	45143	19340–55048	48555	34517–54166
Cul o 9	11495	52–51561	42839	7787–55000	41388	5875–56877
Cul o 10	**5356** ^**b**^	107–34307	13292	4276–44897	**23654** ^**b**^	8690–55174
Cul o 11	39026	15278–47110	31165	8427–48983	41621	5414–54109
Cul o 12	445	0–1978	1041	137–1863	1277	190–13677
Cul o 13	1451	123–45650	3198	479–49726	22463	850–48735
Cul o 14	197	0–2214	56	0–488	134	0–2295
Cul o 15	96	0–4396	302	0–6080	348	13–5898
Cul n 1	597	0–41966	1349	780–48398	4210	399–47084
Cul n 2	239	0–4793	192	0–673	230	29–1408
Cul n 3	3020	844–45049	5824	344–52551	17084	657–54196
Cul n 4	286	45–48935	751	0–17197	2001	121–36137
Cul n 5	118	0–2626	47	2–2277	171	1–4777
Cul n 6	704	0–12758	454	150–1788	1270	412–38382
Cul n 7	1179	0–3069	234	0–1490	421	0–7609
Cul n 8	2293	737–7875	2230	114–3681	1073	331–13366
Cul n 9	2674	305–7540	1190	423–15522	3512	402–47688
Cul n 10	1252	58–12871	1153	372–49574	1835	164–45219
Cul n 11	138	0–28577	1184	60–24667	1539	51–42727

Effect of IBH duration on median IgE levels (in fluorescence arbitrary units) to *Culicoides* r-allergens in horses living in Sweden. Same superscript letters indicate statistically significant differences (P ≤ .05) in Kruskal-Wallis Z-value test (Dunn’s test) with Bonferroni correction for multiple comparisons.

## Discussion

The aim of this study was to identify the primary sensitizing *Culicoides* allergens for equine insect bite hypersensitivity in horses exported from Iceland to continental Europe. Therefore, allergen-specific serum IgE was measured with a newly developed protein microarray [10, 22] which includes a comprehensive panel of 16 Cul o and 11 Cul n r-allergens. As previous studies have shown that allergens derived from *Culicoides* species found in the horses’ environment (i.e. *Culicoides obsoletus*) are more relevant for IBH than laboratory-bred species (i.e. *Culicoides nubeculosus*) [14, 25], it was crucial to test a large panel of Cul o r-allergens. The findings of this study indicate a rise in *Culicoides*-specific IgE concomitant with the initial onset of clinical signs of IBH, and that there is not a single, but many primary sensitizing *Culicoides* allergens. This confirms initial investigations, in which only three Cul o allergens and 10 Cul n r-allergens were included and a smaller number of horses were tested [19]. In the present study, seven major primary sensitizing *C*. *obsoletus* r-allergens were identified, namely Cul o 8, Cul o 11, Cul o 2P, Cul o 7, Cul o 1P, Cul o 13 and Cul o 10. These r-allergens bound IgE in 51 to 78% of the sera at T_IBH_. Two further r-allergens, Cul o 3 and Cul o 9 also appear to be very important at the start of sensitization as they bound IgE in nearly 50% of sera. All these r-allergens, with the exception of Cul o 13, had also previously been identified as those most relevant for IBH in horses from various breeds independent of their origin [10]. Our study indicates that Cul o 13, a D7-related protein, is an important r-allergen in horses exported from Iceland, as >50% of the horses had positive IgE values to this allergen at T_IBH_. Similarly, in the Swedish horse group, IBH horses exported from Iceland had significantly higher IgE levels to this r-allergen than those born in continental Europe. Additionally, a further 10 r-allergens bound IgE more frequently in sera from IBH horses at T_IBH_ compared to the H group, however sero-positivity rates were lower (18–47%, p<0.05).

An overall increase in allergen-specific IgE Ievels was observed in the second year of IBH symptoms (i.e. between T_IBH_ and T_IBH_+1), although this mostly did not reach statistical significance. Conversely, the number of *Culicoides* r-allergens that horses get sensitized to was not found to increase between T_IBH_ and T_IBH_+1.

The highest increase in serum IgE concentrations occurred between T_IBH_-1 and T_IBH_. At T_IBH_-1 the IBH group did not usually differ significantly from the H group or from the unexposed horses. The sero-positivity rate in the H group was somewhat higher than reported by Novotny et al. [10]. This might be due to the fact that nearly none of the H horses in that study were imported from Iceland. Previous studies showed some degree of sensitization in horses imported from Iceland with a healthy end-point, indicating a mechanism for regulation of this initial sensitization [18]. A small percentage of the unexposed horses had IgE positive values to some of the r-*Culicoides* allergens. This might be explained by endoparasite induced high polyclonal IgE [24, 26] binding nonspecifically, or to cross-reactivity between allergens in *Simulium* and *Culicoides* [11]. *Simulium* are present in Iceland and are the only known insects that bite horses in Iceland.

It will be a great interest to determine in the future whether and which IgG subclasse(s) are associated with a non-allergic immune response to *Culicoides* allergens and have blocking properties [27]. Previous studies have shown that horses exposed to *Culicoides* bites which do not develop IBH are not immunologically ignorant to these antigens but have an antigen specific Th1/Treg immune response [28–30]. Horses have seven IgG subclasses [31] and, unfortunately, reagents to determine each of the seven IgG subclasses individually are still missing. Previous studies have shown that IgG5 and IgG3/5 to some *Culicoides* r-allergen are increased in serum of horses with IBH, sometimes even before onset of IBH and might thus have a predictive value [19, 32]. However, no protective IgG subclass has been identified yet [19].

Data from the horses living in Switzerland and in Sweden were analyzed separately due to confounding factors. The horses in Sweden had been exposed to *Culicoides* for a longer time period than the Swiss group, so it is unknown whether time of exposure, type or quantity of insects were responsible for the higher degree of IgE sensitization observed. Analysis of the data from the Swedish group indicates that in horses imported from Iceland several years ago that did not develop IBH, IgE levels were similar to those of H horses born in continental Europe. Hence, for horses not susceptible to *Culicoides* allergy, the provenance is not important. On the other hand, for the susceptible ones within the same breed, there is a clear difference in the sensitization level between IS and N-IS IBH horses: IS horses are sensitized to markedly more *Culicoides* r-allergens and often have higher IgE levels (significant for Cul o 6, Cul o 13 and Cul n 5) than IBH horses born in continental Europe. However, this needs to be confirmed, as the number of IBH horses in the N-IS group was rather small. Nevertheless, this supports our hypothesis that the high degree of sensitization and the high prevalence of IBH in Icelandic horses is not due to the breed itself, but to the presence or absence of *Culicoides* in the environment at early age [4].

Finally, we also investigated whether a longer duration of IBH was associated with sensitization to a higher number of *Culicoides* allergens and/or resulted in higher IgE concentrations to some of the r-allergens. Our data suggests that the number of allergens horses are sensitized to is only slightly higher in horses affected with IBH for many years (>7) than in those affected for a shorter period of time (≤ 7years) (median = 18 versus 15 r-allergens, respectively, ns). Horses with a long history of IBH often had higher *Culicoides*-specific IgE levels, but this difference only reached significance for three r-allergens: Cul o 1P, Cul o 5 and Cul o 10. From our data there is no indication that IgE sensitization decreases over time, even though a reduction of exposure to *Culicoides* through management techniques, such as stabling or use of blankets, is usually done in order to reduce clinical signs of IBH. This is a limitation of this part of the study, as, beside the relatively small groups available to evaluate effects of duration of the disease, treatments could not be accounted for. Furthermore, because of the individual sensitization pattern, a longitudinal study would be more suitable to evaluate such effects.

In conclusion, this study demonstrates that there is no single primary sensitizing *Culicoides* r-allergen, but that horses become sensitized simultaneously to multiple *Culicoides* allergens. This indicates that IgE-reactivity is probably due to co-sensitization as oppose to cross-reactivity between *Culicoides* allergens. The study has enabled the identification of the most relevant primary sensitizing allergens for IBH in horses exported from Iceland to continental Europe. This is a first important step towards the development of preventive allergen immunotherapy for IBH.

## Supporting information

S1 Table Median serum IgE levels to *Culicoides* recombinant (r-)allergens. Median serum IgE levels in fluorescence arbitrary units to *Culicoides* recombinant (r-) allergens in horses imported from Iceland to Switzerland that developed insect bite hypersensitivity (IBH) or remained healthy (H), and in horses living in Iceland (unexposed). Serum samples were taken the summer of clinical onset of IBH (T_IBH_) and at the corresponding time in the H group. Same superscript letters indicate statistically significant differences in Kruskal-Wallis Z-value test (Dunn’s test) with Bonferroni correction for multiple comparisons.
(DOCX)

## List of abbreviations

AIT: allergen-specific immunotherapy
Alt a 1: recombinant mould allergen *Alternaria alternate 1*
CN-TE: *Culicoides nubeculosus* thorax extract
CO-WBE: whole body extract from *Culicoides Obsoletus* group midges
cP: corrected p-value
Cul n: *Culicoides nubeculosus*
Cul o: *Culicoides obsoletus*
Der f: house dust mite extract (*Dermatophagoides farinae*)
FAU: fluorescence arbitrary unit
H: healthy
IBH: insect bite hypersensitivity
IS: born in Iceland and exported to Sweden
N-IS: born in Sweden and living in Sweden
ns: not significant
ROC: Receiver Operator Characteristic
SV-WBE: black fly extract (*Simulium vittatum*)
T_IBH_: summer of first clinical signs of IBH
T_IBH_+1: year following summer of first clinical signs of IBH, i.e. 2^nd^ summer with clinical signs of IBH
T_IBH_-1: year preceding summer of first clinical signs of IBH
unexposed: horses living in Iceland, not exposed to *Culicoides*
