# Supplementary material for: First clinical expression of equine insect bite hypersensitivity is associated with co-sensitization to multiple Culicoides allergens
Source: PLoS One. 2021 Nov 15;16(11):e0257819. doi: 10.1371/journal.pone.0257819 (PMC8592417; doi:10.1371/journal.pone.0257819)
Supplement: S1 Table — Median serum IgE levels in fluorescence arbitrary units to Culicoides recombinant (r-) allergens in horses imported from Iceland to Switzerland that developed insect bite hypersensitivity (IBH) or remained healthy (H), and in horses living in Iceland (unexposed). Serum samples were taken the summer of clinical onset of IBH (TIBH) and at the corresponding time in the H group. Same superscript letters indicate statistically significant differences in Kruskal-Wallis Z-value test (Dunn’s test) with Bonferroni correction for multiple comparisons. (DOCX) [file pone.0257819.s001.docx]

**Table S1. Median serum IgE levels to *Culicoides* recombinant (r-)allergens**

| **allergen name** | **unexposed** | | **H** | | | **IBH** | | **Kruskal-Wallis** |
| --- | --- | --- | --- | --- | --- | --- | --- | --- |
|  | **median** | **range** | **median** | | **range** | **median** | **range** | **p** |
| Cul o 1P | **28^c^** | 0 - 4315 | **1184^b^** | 0 - 23452 | | **20208^b,c^** | 0 - 60978 | <0.00001 |
| Cul o 2 | **72^a,c^** | 0 - 1076 | **24^a^** | 0 - 177 | | **30^c^** | 0 - 1052 | <0.01 |
| Cul o 2P | **52^c^** | 0 - 504 | **5^b^** | 0 - 2808 | | **1430^b,c^** | 0 - 23064 | <0.00001 |
| Cul o 3 | **141^c^** | 31 - 1680 | **260^b^** | 0 - 6718 | | 1034^b,c^ | 0 - 18971 | <0.00001 |
| Cul o 3P | **255^a,c^** | 0 - 4007 | **10^a^** | 0 - 1640 | | 84^c^ | 0 - 2390 | <0.01 |
| Cul o 5 | **243^a,c^** | 0 - 2828 | **1849^ab^** | 0 - 13740 | | **4997^b,c^** | 44 - 55803 | <0.00001 |
| Cul o 6 | **149^a^** | 0 - 235 | **30^ab^** | 0 - 385 | | **94^b^** | 0 - 3603 | <0.0001 |
| Cul o 7 | **133^c^** | 28 - 1093 | **182^b^** | 0 - 3423 | | **2870^b,c^** | 0 - 53996 | <0.00001 |
| Cul o 8 | **0^c^** | 0 - 3373 | **1096^b^** | 0 - 54334 | | **46715^b,c^** | 0 - 60605 | <0.00001 |
| Cul o 9 | **78^c^** | 6 - 1417 | **52^b^** | 0 - 5369 | | **1591^b,c^** | 0 - 61076 | <0.00001 |
| Cul o 10 | **0^c^** | 0 - 259 | **0^b^** | 0 - 4056 | | **753^b,c^** | 0 - 48437 | <0.00001 |
| Cul o 11 | 1397^a,c^ | 412 - 7299 | **5015^ab^** | 0 - 21495 | | **29477^b,c^** | 1035 - 58052 | <0.00001 |
| Cul o 12 | 10 | 0 - 1181 | **4^b^** | 0 - 3086 | | **114^b^** | 0 - 8144 | <0.001 |
| Cul o 13 | **425^c^** | 67 - 7454 | **1094^b^** | 0 - 8641 | | **6641^b,c^** | 61 - 53796 | <0.00001 |
| Cul o 14 | 29 | 0 - 733 | 0 | 0 - 1300 | | 29 | 0 - 1062 | ns |
| Cul o 15 | 73 | 0 - 1408 | 83 | 0 - 3673 | | 173 | 0 - 5573 | 0.07 |
| Cul n 1 | **0^c^** | 0 - 532 | 399 | 0 - 11833 | | **474^c^** | 0 - 40387 | <0.05 |
| Cul n 2 | 40 | 0 - 210 | **3^b^** | 0 - 428 | | **63^b^** | 0 - 3523 | <0.05 |
| Cul n 3 | **286^a,c^** | 0 - 1816 | **926^ab^** | 0 - 18607 | | **2767^b,c^** | 0 - 59729 | <0.00001 |
| Cul n 4 | **12^c^** | 0 - 677 | **1^b^** | 0 - 3600 | | **531^b,c^** | 0 - 37001 | <0.00001 |
| Cul n 5 | **49^a^** | 0 - 153 | **4^ab^** | 0 - 181 | | **94^b^** | 0 - 4296 | <0.00001 |
| Cul n 6 | 0 | 0 - 544 | 0 | 0 - 1304 | | 45 | 0 - 21674 | 0.05 |
| Cul n 7 | 113 | 0 - 970 | 387 | 0 - 5888 | | 391 | 0 - 5495 | ns |
| Cul n 8 | **290^c^** | 0 - 3398 | **870^b^** | 0 - 6783 | | **1778^b,c^** | 0 - 35119 | <0.0001 |
| Cul n 9 | 336 | 167 - 2441 | 482 | 0 - 6098 | | 701 | 0 - 29987 | <0.05 |
| Cul n 10 | 445 | 26 - 2626 | 442 | 0 - 7390 | | 708 | 0 - 55321 | ns |
| Cul n 11 | 92 | 0 - 947 | 92 | 0 - 8205 | | 348 | 0 - 9431 | ns |
| CO-WBE | **290^c^** | 79 - 763 | **388^b^** | 0 - 3280 | | **1016^b,c^** | 0 - 7443 | <0.00001 |
| CN-TE | 328 | 129 - 1012 | **251^b^** | 0 - 4330 | | **473^b^** | 0 - 4016 | <0.001 |
| SV-WBE | 457 | 64 - 1650 | **299^b^** | 0 - 2276 | | **616^b^** | 0 - 6537 | <0.001 |
| Alt a 1 | **102^a,c^** | 22 - 303 | **24^a^** | 0 - 239 | | 5^c^ | 0 - 482 | <0.00001 |
| Der f | **670^a^** | 206 - 5720 | 412 | 0 - 11492 | | **293^a^** | 0 - 15242 | <0.05 |

Median serum IgE levels in fluorescence arbitrary units to *Culicoides* recombinant (r-) allergens in horses imported from Iceland to Switzerland that developed insect bite hypersensitivity (IBH) or remained healthy (H), and in horses living in Iceland (unexposed). Serum samples were taken the summer of clinical onset of IBH (T_IBH_) and at the corresponding time in the H group. Same superscript letters indicate statistically significant differences in Kruskal-Wallis Z-value test (Dunn's test) with Bonferroni correction for multiple comparisons.
