# Supplementary material for: First clinical expression of equine insect bite hypersensitivity is associated with co-sensitization to multiple Culicoides allergens
Source: PLoS One. 2021 Nov 15;16(11):e0257819. doi: 10.1371/journal.pone.0257819 (PMC8592417; doi:10.1371/journal.pone.0257819)
Supplement: Attachment — Submitted filename: PONE-D-21-15096 .docx [file pone.0257819.s002.docx]

**PONE-D-21-15096**

The paper provides novel information on the development of Equine Insect bite hypersensitivity (IBH) that could be important for the establishment of therapy and prevention of this type of allergy in the future..

The study includes a large number of horses inside every group studied (224 in total) and a control group. A follow-up of three years (enough to obtain good conclusions about the development of the IBH) has been made.

The statistical analyse is well described and is adequate for the data (not normally distribution) and number of animals.

The technique used for the evaluation of the IgE microarray proteins allows the detection of antigenic protein molecules with more precise results than other immunoassay systems used to date and is well described by the authors.

The paper manages to achieve the proposed objectives.

The discussion could be enriched with a broader analysis of the knowledge situation of the IBH so far, but it is understood that the authors have not wanted to enter into comparative aspects because the microarray technique is totally different and much more specific than others used up to now.

I consider that it is suitable to be published.

One error should be corrected on line 40 (same as in the first presentation summary): (<4 years, 4-7 years, **> 7 years**).
